# Pyrite High-Entropy Sulfides for Bifunctional Oxygen Electrocatalysis and High-Performance Zinc-Air Batteries

**DOI:** 10.1021/acsomega.5c12740

**Published:** 2026-02-19

**Authors:** Tuncay Erdil, Nazlican Uysal, Zeynep Ilgın Yüceer, Cagla Ozgur, Uygar Geyikci, Cigdem Toparli

**Affiliations:** † Department of Metallurgical and Materials Engineering, 52984 Middle East Technical University, 06800 Ankara, Türkiye; ‡ Energy Storage Materials and Devices Research Center (ENDAM), Middle East Technical University, Ankara 06800, Türkiye

## Abstract

Zinc-air batteries are limited by sluggish, reversible oxygen electrocatalysis at the air cathode. High-entropy sulfides (HESs) are introduced as bifunctional oxygen catalysts, leveraging multication disorder to create robust, tunable active sites. Three pyrite-type, single-phase compositions: HES-TM [(FeNiCoCrMn)­S_2_], HES-CuTi [(FeNiCoCuTi)­S_2_], and HES-Co0.4 [(FeNiCo_0.4_CrMn)­S_2_], exhibit homogeneous elemental distributions and nanoscale particles. Among them, HES-TM delivers the strongest overall bifunctionality (0.94 V), combining lower OER overpotential with more favorable ORR polarization. Koutecký-Levich analysis indicates mixed pathways consistent with partially four-electron ORR. XPS reveals predominantly Co^3+^ with mixed Ni and Fe valence, while HES-TM shows a higher sulfide (S_2_^2–^) fraction with a thin SO*_x_* surface layer, signatures of stronger metal–sulfur coordination, and optimized *OH/*OOH binding. Despite higher conductivity in HES-CuTi, activity trends confirm that active-site chemistry, not conductivity alone, governs performance. In full zinc-air cells, HES-TM reduces the charge–discharge voltage gap and enhances power and energy delivery, consistent with its low bifunctional index. These results position disorder-engineered pyrite HESs as cost-effective, scalable cathodes and provide design rules that link surface sulfur chemistry and mixed metal valence to reversible oxygen electrocatalysis.

## Graphical abstract

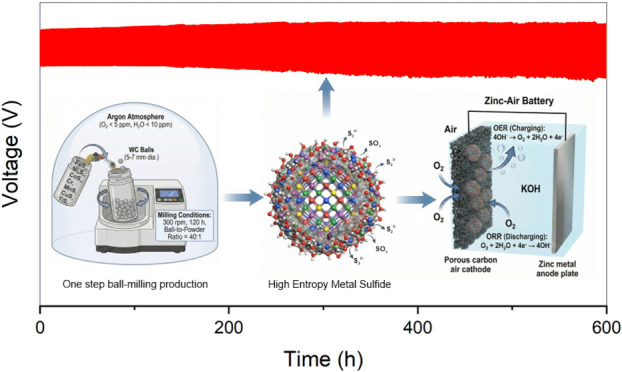

## 1 Introduction

The growing global demand for sustainable and high-efficiency energy conversion and storage systems has accelerated the search for next-generation rechargeable batteries beyond traditional lithium-ion technologies. As energy storage plays a pivotal role in ensuring energy security and enabling the transition toward low-carbon energy systems, the development of advanced electrochemical storage technologies such as zinc-air batteries (ZABs) has become increasingly critical.^1–5^ Among the various candidates, ZABs have attracted tremendous attention owing to their high theoretical energy density (1218 Wh kg^–1^), cost-effectiveness, intrinsic safety, and environmental benignity.^1,6–9^ However, the sluggish kinetics of the oxygen reduction reaction (ORR) during discharge and the oxygen evolution reaction (OER) during charge remain the major bottlenecks that limit round-trip efficiency and long-term stability.^2,3,10^ Conventional noble-metal catalysts such as Pt/C and RuO_2_ exhibit excellent activity but suffer from high cost, limited durability, and poor bifunctionality under realistic cycling conditions.^11–13^ Therefore, the rational design of earth-abundant, highly active, and durable bifunctional catalysts is crucial for advancing practical ZAB applications.

One strategy is to employ high-entropy materials (HEMs).^14–18^ In such systems, the high mixing entropy (Δ*S*_conf_ ≥ 1.5R) promotes solid-solution formation, enhances lattice distortion, and generates abundant defect sites, collectively improving mechanical robustness and electronic versatility.^10,12,19–21^ These entropy-driven effects have been exploited to tune thermodynamic stability, ionic transport, and surface redox properties, providing an unprecedented degree of compositional tunability for energy devices.^22,23^ On the other hand, conventional transition-metal sulfides have already shown promising activity and stability toward the OER due to their intrinsic electronic and structural tunability.^24,25^ As a further step, the integration of sulfur in HEM systems has given rise to a new subclass: high-entropy sulfides (HESs).^26–28^ Sulfur introduces additional degrees of freedom by altering metal-anion interactions, modifying electronic density around catalytic centers, and facilitating enhanced electrical conductivity compared to oxide analogues.^29–31^ Using multiple-elemental synergy to modify the charge state in metal sulfides and tune the catalyst-adsorbate interaction has good potential for increasing catalytic activity.^29–32^ Recent studies further revealed that high-entropy sulfides can undergo self-reconstruction during OER, forming active metal (oxy)­hydroxides while retaining beneficial sulfate species that promote charge transfer and long-term stability.^30,32^ The combination of multiple transition metals in a sulfide matrix results in rich local chemical environments, enabling broad distribution of active sites and tunable adsorption energies.^29–31^ These features are particularly advantageous for bifunctional ORR/OER catalysis in rechargeable ZABs, where activity, selectivity, and long-term durability must be simultaneously optimized.^30–34^

Herein, we report the synthesis and electrochemical evaluation of a high-entropy sulfide catalyst, (Fe, Ni, Co, Cr, Mn, Cu, Ti)­S_*x*_ system, as a bifunctional oxygen catalyst for rechargeable Zn-air batteries. The synergistic coupling among five transition-metal species and sulfur anions enables optimized electronic structure and abundant redox-active sites, enhancing both OER and ORR kinetics. As a cathode material in a Zn-air battery, the catalyst delivers a high capacity of 675.9 mAh, a peak power density of 88 mW cm^–2^, and maintains stable charge–discharge operation for more than 25 days. These results demonstrate that entropy engineering effectively couples thermodynamic stabilization with kinetic enhancement, providing both high activity and exceptional durability. This work not only provides fundamental insights into entropy-driven electronic modulation in multication sulfides but also establishes new possibilities for the rational design of advanced catalyst materials that can meet the demanding requirements of next-generation energy storage systems. Looking ahead, the exceptional performance and versatility of these HES catalysts suggest promising opportunities for their integration into flexible and rechargeable ZAB configurations.

## 2 Experimental Section

### 2.1 Materials Synthesis

The high-entropy sulfide (HES) catalysts: (FeNiCoCrMn)­S_2_, (FeNiCoCuTi)­S_2_, and (FeNiCo_0.4_CrMn)­S_2_, were synthesized via a mechanochemical process. First, stoichiometric mixtures of precursor powders (FeS_2_, Ni_3_S_2_, CoS_2_, Cr, MnS, CuS, and TiS_2_, ≥99% purity) were combined in a high-energy planetary ball mill (Retsch PM400) using WC vials and balls (5–7 mm diameter) under an argon atmosphere (O_2_ < 5 ppm, H_2_O < 10 ppm). Then, the milling was conducted at 300 rpm for 120 h with a ball-to-powder ratio of 40:1.

### 2.2 Materials Characterization

The crystal structures of the synthesized high-entropy sulfides (HESs) were characterized by powder X-ray diffraction (XRD, Bruker D8 Advance) using Cu Kα radiation (λ = 1.5406 Å) in a 2θ range of 20–90°. The obtained patterns were compared with standard ICDD reference data to confirm phase purity. The morphology and microstructure of the HESs were examined using a high-resolution field-emission scanning electron microscope (FEI Quanta 400 FEG) and a high-resolution transmission electron microscope (JEOL JEM-2100F, 200 kV). High-resolution transmission electron microscopy (HRTEM) was employed to obtain lattice-resolved images, high-angle annular dark-field (HAADF) micrographs, the corresponding energy-dispersive spectroscopy (EDS) elemental mapping, and selected area electron diffraction (SAED) patterns. X-ray photoelectron spectroscopy (XPS, Thermo Scientific K-α) with Al Kα radiation was used to investigate the surface chemical states of the metal and sulfur species, with all spectra calibrated against the C 1s peak at 284.8 eV.

### 2.3 Electrochemical Measurements

#### 2.3.1 Half-Cell Tests

The electrocatalytic activity of the high-entropy sulfide (HES) materials was first evaluated using a standard three-electrode RDE setup (BASI) connected to a GAMRY Reference 3000 potentiostat. The system consisted of an Ag/AgCl reference electrode (3 M KCl), platinum wire counter electrode, and glassy carbon working electrode (5 mm diameter). Catalyst inks were prepared by dispersing 10 mg HES material with 5 mg Super-P carbon and 50 μL Nafion (5 wt %) in 2 mL ethanol, followed by 30 min sonication. A 10 μL aliquot was drop-cast onto the GCE (loading: 0.4 mg cm^–2^) and air-dried.

All measurements were conducted in O_2_-saturated 1 M KOH at 25 °C. Potentials were converted to the RHE scale (*E*_vsRHE_ = *E*_vs Ag/AgCl_ + 0.059 × pH + 0.1976) and *iR*-corrected. LSV was performed from 0.2 to 1.1 V vs Ag/AgCl at 10 mV s^–1^ with a rotation rate of 1600 rpm. EIS measurements used a 10 mV AC amplitude from 10^5^ to 10^–2^ Hz at a near overpotential voltage of 1.6 V.

### 2.4 Zinc-Air Battery Assembly

For practical zinc-air battery (ZAB) testing, the HES catalysts were incorporated into air cathodes by loading 2 mg cm^–2^ of active material onto hydrophobic gas diffusion layers. The anode consisted of polished zinc foil treated with 0.1 M HCL to ensure a clean, active surface. The electrolyte solution comprised 6 M KOH with 0.2 M zinc acetate (Zn­(OAc)_2_) additive.

Battery performance was evaluated using a custom-designed cell configuration. Galvanostatic discharge–charge cycling tests were performed at a current density of 5 mA cm^–2^, with each cycle consisting of 5 min of discharge followed by 5 min of charge. Their power densities were calculated using discharge polarization between 1.5–0.1 V. Batteries were fully discharged at 5, 10, and 20 mA cm^–2^ current densities to find the specific capacities. During the rate capability test, the discharge current density was progressively increased from 0 to 20 mA cm^–2^ and subsequently decreased back to 0 mA cm^–2^, while the corresponding cell voltage was continuously recorded.

## 3 Results and Discussion

### 3.1 Structural and Morphological Analysis of HES Catalysts

High-entropy materials (HEMs) are stabilized by a high configurational entropy (*S*_config_) arising from the random distribution of five or more principal cations over a single crystallographic sublattice (e.g., the A- or B-site in perovskites, or the tetrahedral/octahedral sites in spinels). This random, multicomponent occupation greatly expands compositional space and enables synergistic cation–cation interactions that are advantageous for catalysis. For nonmetallic systems (e.g., oxides, sulfides, chlorides), the molar configurational entropy can be estimated by summing the mixing entropy of each crystallographic sublattice. A commonly used expression is

$$S_{\mathrm{config}}=R\left(\sum_{i\in \mathrm{cations}}x_{i}\ln x_{i}+\sum_{j\in \mathrm{cations}}y_{j}\ln y_{j}\right)$$

Where *x_i_* and *y_j_* are the mole fractions of the cation sublattice and the anion sublattice ion species, respectively (with ∑*x_i_* = 1and ∑*y_j_* = 1); *R* is the universal gas constant. Following an empirical convention, materials are classified by their configurational entropy (*S*_config_) as follows: high-entropy if *S*_config_ is greater than or equal to 1.5*R*; medium-entropy if *S*_config_ is between 1.0 and 1.5*R*; and low-entropy if *S*_config_ is less than 1.0*R*.

The X-ray diffraction (XRD) patterns in Figure 1a indicate that all sulfide samples crystallize in the pyrite-type structure (space group *Pa*3̅). The nearly identical profiles across compositions confirm a uniform crystal structure among the high-entropy sulfides (HESs) despite their elemental variation. No secondary oxide or hydroxide phases of the constituent elements (Fe, Ni, Co, Cr, Mn, Cu, Ti) are detected for HES-TM, HES-CuTi, and HES-Co0.4. A single weak extra reflection at 2θ ≈ 48.5° (marked by an asterisk) appears in all patterns and matches the (101) plane of WC, originating from abrasion of the tungsten-carbide milling media. ICP-OES results in Table S1 show that the elemental ratios of the HESs are unaffected; therefore, this trace WC impurity is considered extrinsic and is not expected to influence the catalytic performance. Rietveld refinements (Figures 1b and S1) further confirm the pyrite structure (*Pa*3̅). The refined lattice parameters are *a* = 5.451 Å, 5.439 Å, and 5.447 Å for HES-TM, HES-CuTi, and HES-Co0.4, respectively. As expected, compositions with a smaller average cation radius exhibit a slight lattice contraction, reflected in decreased lattice constant and unit-cell volume.

**Figure 1:**
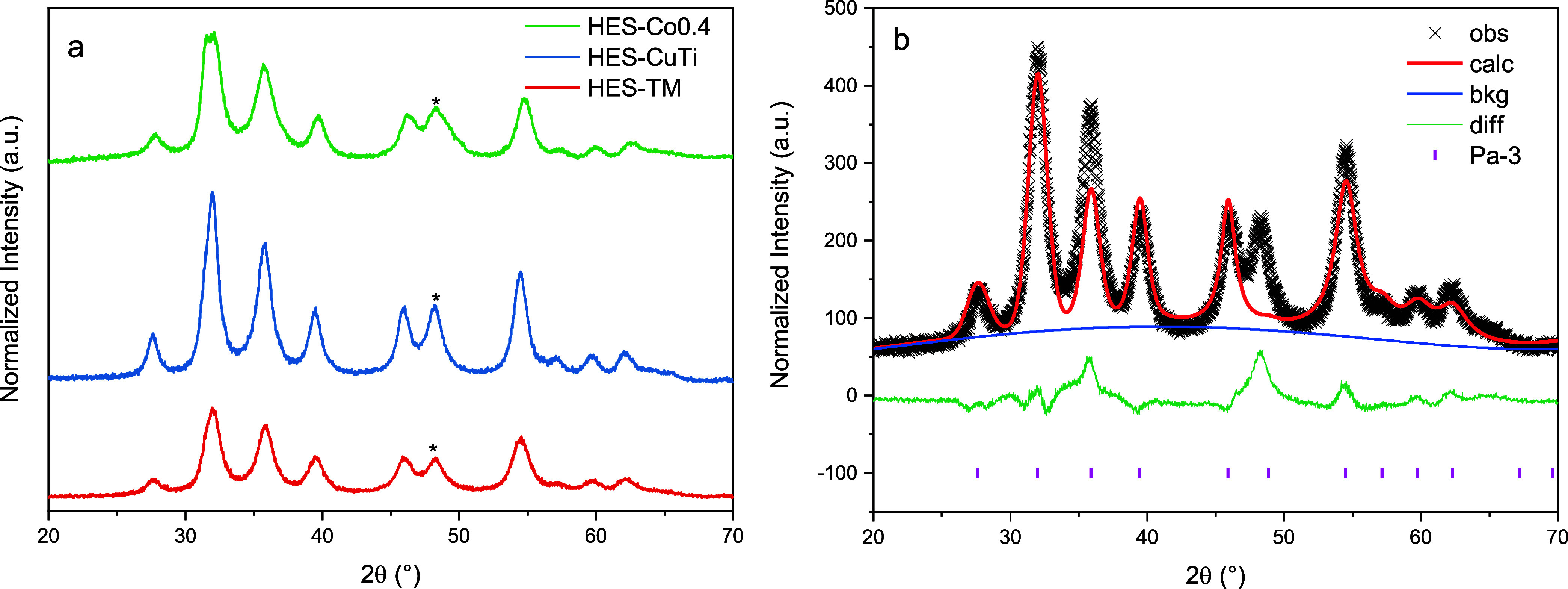
(a) XRD patterns of high entropy sulfide series, (b) Rietveld refinement profile of HES-TM.

Bright-field transmission electron microscopy (TEM) images and the corresponding selected-area electron diffraction (SAED) pattern of HES-TM are shown in Figure 2a. The imaged particle has a characteristic size of ∼300–500 nm, in line with SEM observations. The SAED pattern consists of sharp, well-defined reflections that are indexed to the cubic pyrite structure (space group *Pa*3̅), with no extra spots or diffuse rings indicative of secondary or amorphous phases. High-resolution TEM (HRTEM) reveals clear lattice fringes across extended regions; the measured interplanar spacing of *d* ≈ 0.146 nm corresponds to the (312) planes, consistent with *Pa*3̅ symmetry (Figure 2d). Taken together with the XRD Rietveld refinements reported above, these results confirm that HES-TM is a single-phase, homogeneous solid solution with a cubic pyrite structure. An analogous set of TEM characterizations was performed for HES-CuTi and HES-Co0.4 (Figure 2b,e,2c,f, respectively). Both powders exhibit particle sizes around 300–500 nm and display SAED patterns that index cleanly to *Pa*3̅ symmetry, in agreement with the Rietveld-refined XRD results. HRTEM further corroborates the cubic pyrite structure, with measured *d*-spacings of ∼0.144 nm (HES-CuTi) and ∼0.145 nm (HES-Co0.4) for the (312) planes. Across multiple regions and particles, the fringe continuity and absence of extra diffraction features support good crystallinity and the lack of detectable secondary phases. Overall, the TEM/SAED/HRTEM findings are fully consistent with the XRD analysis, reinforcing that all three compositions form single-phase, pyrite-type high-entropy sulfides.

**Figure 2:**
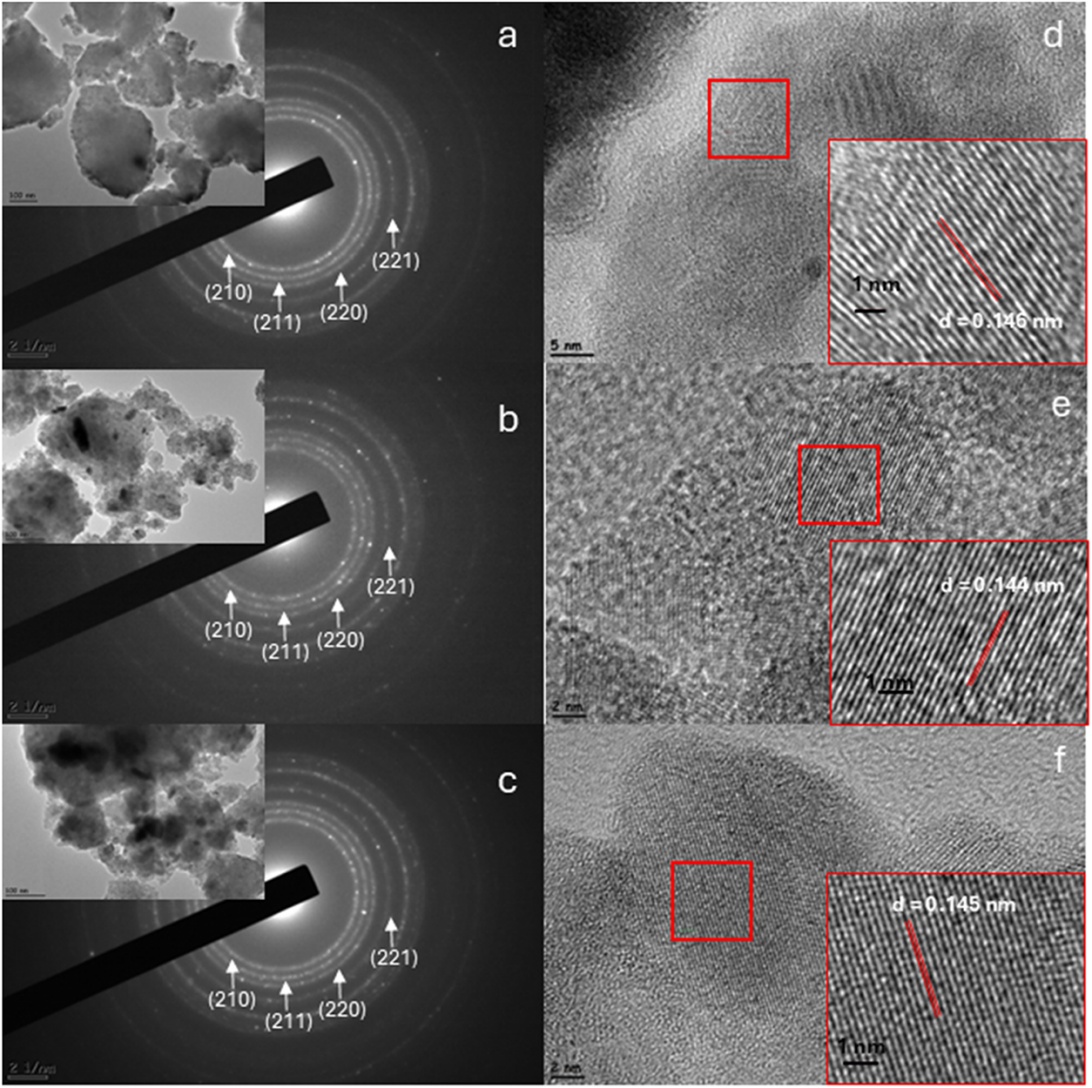
SAED patterns of (inset bright field images) (a) HES-TM, (b)­HES-CuTi, (c) HES-Co0.4, and HRTEM images of (d) HES-TM, (e) HES-CuTi, (f) HES-Co0.4.

The morphologies and elemental distributions were examined by SEM and EDS elemental mapping. Figures 3 and S2 show SEM micrographs of the HES powders. All compositions exhibit similar particle morphologies characteristic of the ball-milling process, with a relatively narrow particle-size distribution of ∼300–500 nm. The accompanying EDS maps indicate a uniform, homogeneous spatial distribution of the constituent elements across the particles (within the spatial resolution and detection limits of EDS). No tungsten carbide (WC) segregation or WC-rich agglomerates are observed; any WC introduced during milling appears finely dispersed rather than forming bulky debris as shown in Figure S3. This uniform distribution trend is consistent across all synthesized HES samples.

**Figure 3:**
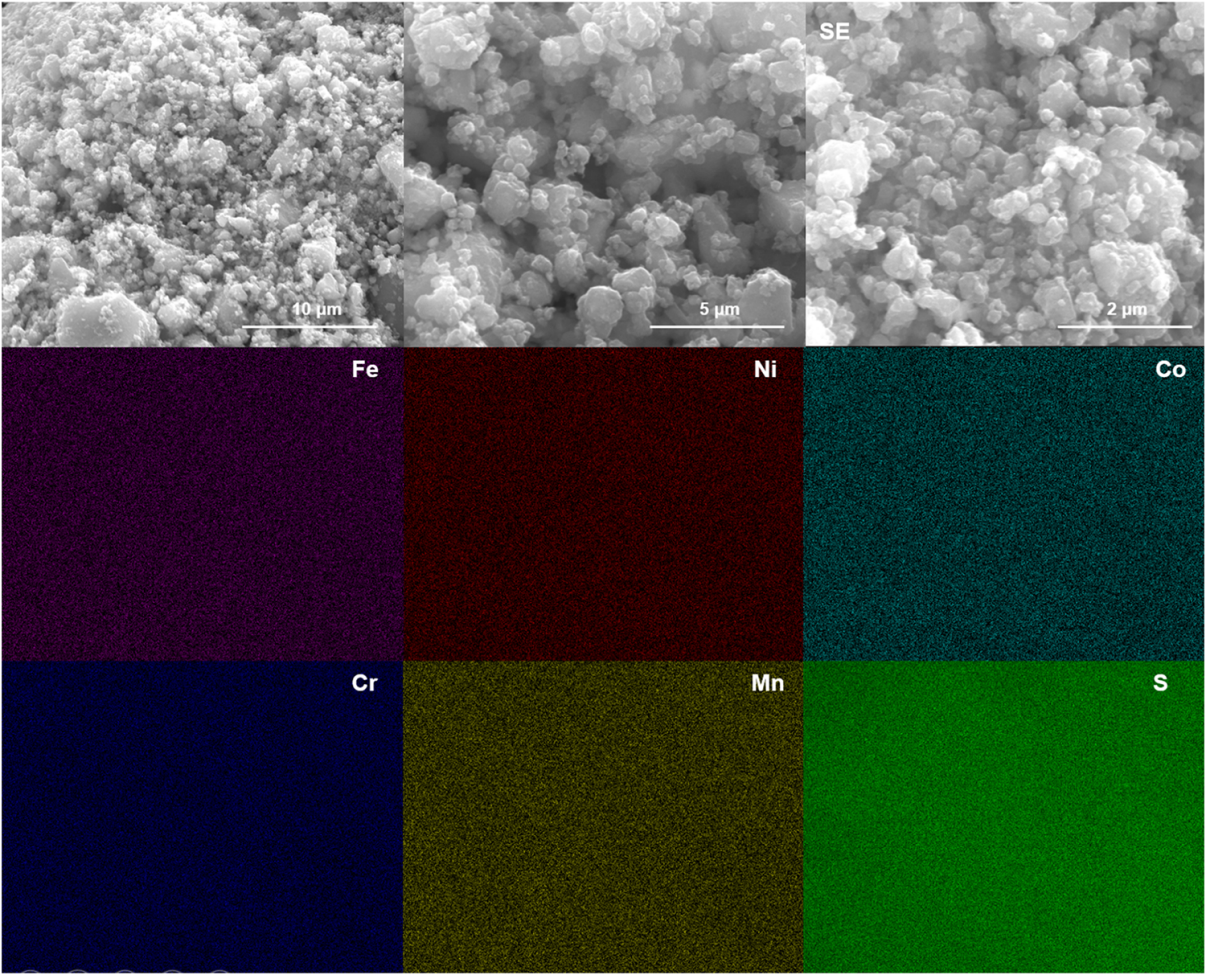
SEM and EDS elemental mapping of HES-TM.

### 3.2 Electronic Structure of the Catalysts

The surface chemistry and oxidation states of the high-entropy sulfides (HESs) were analyzed by X-ray photoelectron spectroscopy (XPS). The XPS survey spectra for each composition are shown in Figure 4. The surveys indicate surface signals from S, Fe, Ni, Co, Cr, and Mn for all HESs except HES-CuTi; in HES-CuTi, Cu and Ti are present instead of Cr and Mn, consistent with its nominal composition.

**Figure 4:**
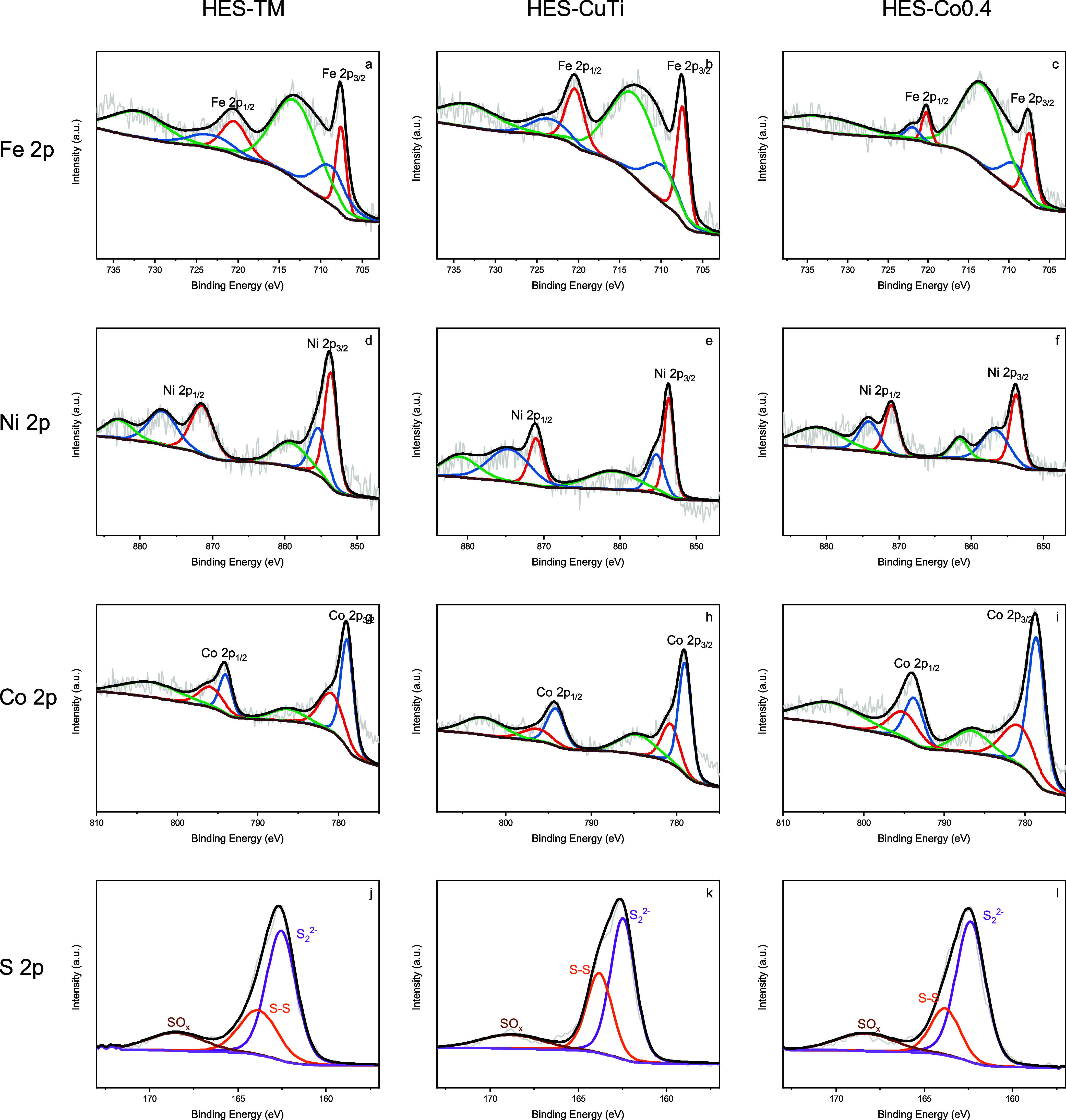
Fe 2p core level spectra of (a) HES-TM, (b) HES-CuTi, (c) HES-Co0.4; Ni 2p core level spectra of (d) HES-TM, (e) HES-CuTi, (f) HES-Co0.4; Co 2p core level spectra of (g) HES-TM, (h) HES-CuTi, (i) HES-Co0.4, S 2p core level spectra of (j) HES-TM, (k) HES-CuTi, (l) HES-Co0.4.

The deconvoluted XPS core-level spectra of the transition metals are color-coded in Figure 4 (red: 2+ charge from sulfur interaction, blue: 3+ charge for the element interaction with surface oxygen, green: satellite features). Determining precise oxidation states in these multication sulfides is intrinsically challenging; reported values should be treated as semiquantitative indicators of average oxidation state rather than exact assignments. In a randomly substituted lattice, variations in local coordination and ligand fields introduce chemical-shift dispersion and final-state screening, causing small but meaningful shifts in core-level binding energies. Because phase-pure reference standards for these novel local environments do not exist, absolute oxidation-state calibration is not feasible. Consequently, oxidation states are best discussed as ranges and trends across samples, not as single definitive integers.^35,36^

The Fe 2p envelope was deconvoluted into spin–orbit components (2p_3/2_ and 2p_1/2_), each comprising Fe^2+^ and Fe^3+^ contributions, together with their associated satellite peaks. In the 2p_3/2_ region, peaks at ∼707.5 eV and ∼711.0 eV are assigned to Fe^2+^ and Fe^3+^, respectively. In the 2p_1/2_ region, subpeaks at ∼721 eV (Fe^2+^) and ∼724 eV (Fe^3+^) are observed. The corresponding satellites occur near ∼714 eV (for 2p_3/2_) and ∼731 eV (for 2p_1/2_).^7,37^ Overall, the Fe chemical state across the HES series spans mixed valence between Fe^2+^ and Fe^3+^. Consistent fitting with spin–orbit constraints (2p_1/2_–2p_3/2_ splitting ≈13 eV and an area ratio near 1:2) shows that HES-CuTi exhibits a modestly higher Fe^2+^/Fe^3+^ ratio than the other compositions. This trend is robust and aligns with the compositional differences of HES-CuTi (Cu/Ti substitution), which can stabilize lower Fe valence by altering local crystal-field and electrostatic environments. The relatively pronounced ∼714 eV satellite intensity is consistent with Fe^2+^ in a sulfide environment and with the expected multiplet/charge-transfer (“shake-up”) features arising from Fe 3d-S 3p hybridization in octahedral FeS_6_ units. In such covalent lattices, stronger satellite intensity generally reflects a larger ligand-to-metal charge-transfer contribution and higher Fe–S covalency, which often accompanies an increased Fe^2+^ fraction (as observed for HES-CuTi). This behavior is therefore in line with the chemical-state assignment from the main Fe 2p components and supports the presence of Fe^2+^-rich local environments in the pyrite-type structure.

The Ni 2p spectra were deconvoluted into Ni^2+^ and Ni^3+^ components for both spin–orbit partners. In the 2p_3/2_ region, peaks centered at ∼853.0 eV and ∼856.0 eV are assigned to Ni^2+^ and Ni^3+^, respectively. The 2p_1/2_ features at ∼871.2 eV (Ni^2+^) and ∼875.0 eV (Ni^3+^) show the corresponding splitting, with a 2p_3/2_-2p_1/2_ separation in the expected ∼17–18 eV range. Characteristic shakeup satellites appear near ∼860 eV (2p_3/2_) and ∼879 eV (2p_1/2_).^7^ Across the HES series, all samples exhibit a mixed Ni^2+^/Ni^3+^ chemical state, with Ni^3+^ oxidation state clearly dominating. The moderate satellite intensity is consistent with Ni in a covalent sulfide environment (octahedral NiS_6_ motifs), where ligand-to-metal charge transfer contributes to the line shape. Such mixed valence and Ni–S covalency are typical of conductive sulfides and may support redox flexibility at the catalyst surface.

The Co 2p spectra were deconvoluted into Co^3+^ and Co^2+^ components for both spin–orbit partners. In the 2p_3/2_ region, peaks at ∼778.5 eV and ∼781.0 eV are assigned to Co^3+^ and Co^2+^, respectively. The corresponding 2p_1/2_ features occur at ∼794.0 eV (Co^3+^) and ∼798.0 eV (Co^2+^), giving a 2p spin–orbit separation in the expected ∼15–17 eV range. Shake-up (satellite) features are observed near ∼785 eV and ∼804 eV, consistent with multiplet/charge-transfer effects in Co–S bonding and octahedral CoS_6_ coordination.^38^ Across all HES samples, the Co^3+^ components (≈778.5 and 794.0 eV) are more intense than their Co^2+^ counterparts (≈781 and 798 eV), indicating that surface cobalt is predominantly in the +3 oxidation state due to surface oxidation, distinct from the more mixed-valent behavior noted for Fe and Ni. This Co^3+^-leaning surface speciation, together with the moderate satellite intensity, suggests appreciable ligand-to-metal charge transfer and Co–S covalency, features that can enhance redox flexibility at the catalyst interface.

The S 2p XPS spectra are shown in Figure 4j–l. Deconvolution yields three chemically distinct components, each modeled as a 2p doublet with a fixed 2p_3/2_–2p_1/2_ splitting (∼1.18 eV) and a 2:1 area ratio. The dominant component at ∼162.5 eV (2p_3/2_) is assigned to disulfide (S_2_^2–^) species characteristic of pyrite-type metal sulfides. A higher-binding energy component near ∼164.0 eV corresponds to S–S species (elemental sulfur/polysulfide, S^0^/Sx), while features at ∼168.5–169 eV are attributed to surface sulfur oxides (SO*_x_*, e.g., sulfite/sulfate) formed by mild air exposure.^7,37,38^ From the relative doublet areas, HES-TM and HES-Co0.4 exhibit a higher fraction of sulfide-like S_2_^2–^ and a modestly greater SO*_x_* contribution compared with HES-CuTi, whereas HES-CuTi shows a relatively larger S–S (≈164 eV) component. The higher S_2_^2–^ fraction and reduced S–S signal in HES-TM/HES-Co0.4 indicate more extensive metal–sulfur coordination at the surface (fewer sulfur-terminated domains), consistent with slightly stronger M–S bonding. Concurrently, the enriched SO*_x_* fraction suggests the presence of a thin oxy-sulfide layer produced upon air contact that remains confined to the near-surface. These sulfur-site trends align with the transition-metal XPS results discussed above. Cobalt is predominantly Co^3+^ across all samples, while nickel is mixed Ni^2+^/Ni^3+^ without a clear bias. Iron spans Fe^2+^/Fe^3+^, with HES-CuTi showing a slightly higher Fe^2+^/Fe^3+^ ratio. The comparatively reduced Fe state in HES-CuTi is consistent with its lower SO*_x_* signal and greater S–S contribution, implying less oxidative surface conditioning. By contrast, HES-TM (and to a lesser extent HES-Co0.4) combines stronger M-S coordination (larger S_2_^2–^ fraction) with a modest SO*_x_* presence. Such an oxy-sulfide-modified, metal-bonded surface can facilitate −OH/O* adsorption and electron transfer, rationalizing the lower OER/ORR overpotentials measured for HES-TM (e.g., 397 mV at 10 mA cm^–2^) relative to HES-CuTi/HES-Co0.4. In short, the S 2p and metal 2p analyses together suggest that (i) stronger metal–sulfur bonding (more S_2_^2–^, fewer S–S terminations) and (ii) a thin, electronically coupled SO*_x_* layer synergistically contribute to the enhanced activity and stability of HES-TM compared with the other compositions.

The remaining core-level spectra are provided in the Figure S4. Cr 2p and Mn 2p spectra for (FeNiCoCrMn)­S_2_, (FeNiCoCuTi)­S_2_, and (FeNiCo_0.4_CrMn)­S_2_ are presented in Figure S3a,b. While the Mn 2p spectra were deconvoluted analogously to Fe 2p, Ni 2p, and Co 2p, the Cr 2p spectra of the high-entropy disulfides exhibit two well-defined peaks at ∼576.5 eV and ∼586.0 eV, corresponding to Cr 2p_3/2_ and Cr 2p_1/2_ of Cr^3+^, respectively. The deconvoluted Mn 2p spectra show components at ∼641 eV and ∼644 eV, attributable to Mn^2+^ and Mn^3+^ in the 2p_3/2_ region, with the corresponding 2p_1/2_ features at ∼653 eV (Mn^2+^) and ∼655 eV (Mn^3+^); shakeup satellites appear near ∼646 eV and ∼658 eV, as expected for Mn in sulfide environments.

Cu 2p and Ti 2p spectra of (FeNiCoCuTi)­S_2_ are shown in Figure S3c,d. The Cu 2p doublet is observed at 932.4 eV (2p_3/2_) and 952.4 eV (2p_1/2_). The absence of intense shakeup satellites in the 940–945 eV region supports assignment to Cu^+^ rather than Cu^2+^ in this sulfide matrix. The Ti 2p spectrum exhibits a doublet at 458.9 eV (2p_3/2_) and 464.6 eV (2p_1/2_), consistent with Ti^4+^; given the high binding energies, these features likely arise from a thin surface oxysulfide/oxide layer formed upon air exposure. Overall, these assignments are consistent with the mixed-valence behavior resolved for Fe and Ni and the predominantly Co^3+^ character observed in Co 2p, reinforcing a chemically coherent picture across the transition-metal sublattice.

### 3.3 Electrochemical Activity of High-Entropy Sulfides

The electrocatalytic activity of the HESs was evaluated in a standard three-electrode configuration using a rotating disk electrode (RDE). Linear sweep voltammetry (LSV) was recorded with an Ag/AgCl reference electrode, and all potentials were converted to the reversible hydrogen electrode (RHE) scale. Currents were normalized to the geometric area of the glassy carbon disk (0.196 cm^2^), as shown in Figure 5. At a benchmark current density of 10 mA cm^–2^, the overpotentials (η_10_) of HES-TM, HES-CuTi, and HES-Co0.4 were 310 mV, 440 mV, and 525 mV, respectively. HES-TM thus exhibits a markedly lower overpotential than the other sulfide electrocatalysts, along with a low onset potential and a steep polarization (current–potential) slope, indicative of superior reaction kinetics. This performance may be associated with stronger sulfur-hydroxide interfacial interactions and the intrinsically high electrical conductivity typical of high-entropy sulfides. The result underscores the importance of an equimolar, multication high-entropy composition in promoting synergistic electronic/structural effects and a high density of active sites.

**Figure 5:**
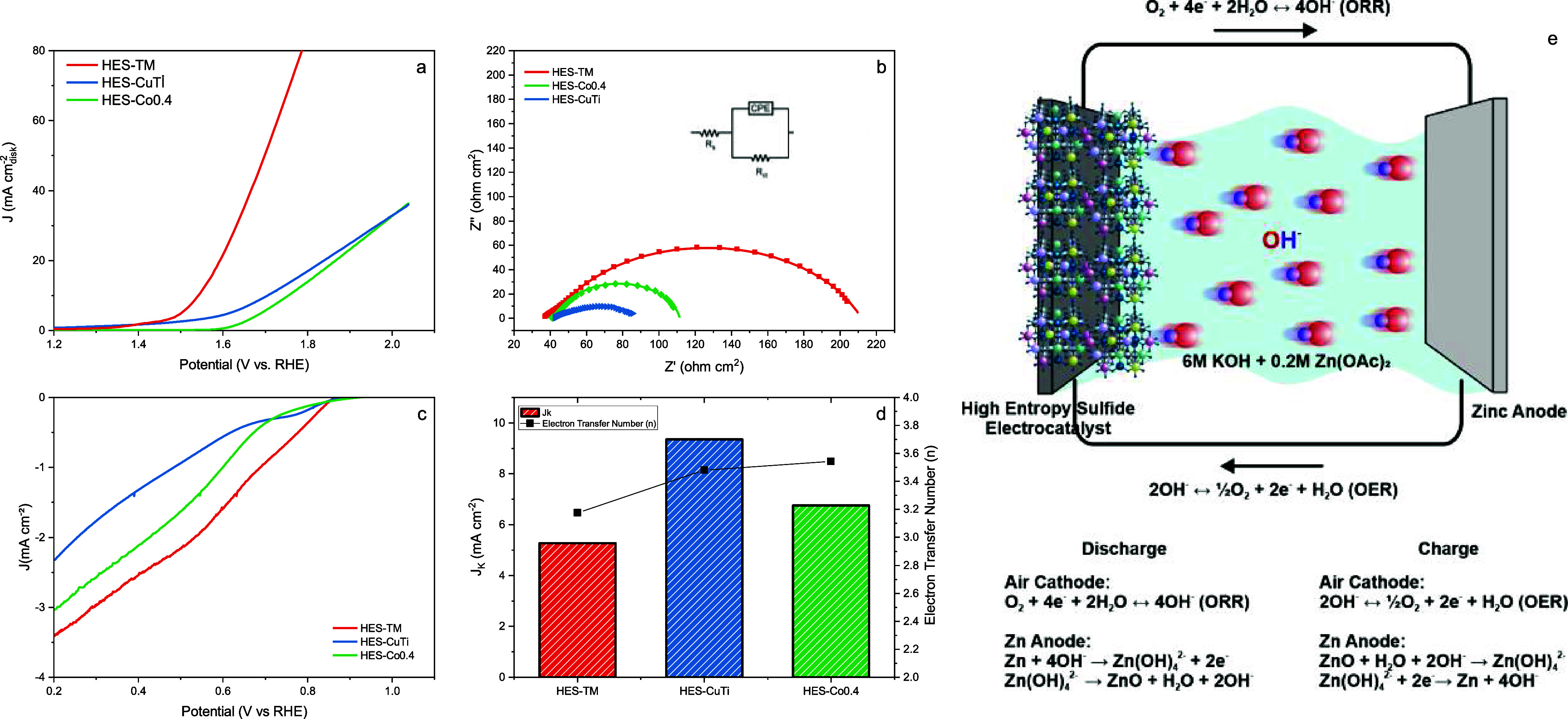
HES-TM, HES-CuTi and HES-Co0.4’s (a) OER activity curves, (b) electrochemical impedance spectroscopy, (c) ORR activity curves, (d) Koutecky–Levich analysis results, (e) schematic illustration of the rechargeable zinc-air battery configuration utilizing the High Entropy Sulfide (HES) air cathode and Zinc anode in 6 M KOH + 0.2 M Zn­(OAc)$_2$ electrolyte, showing the fundamental discharge (ORR) and charge (OER) reaction mechanisms.

Electrochemical impedance spectroscopy (EIS) was performed to probe interfacial charge-transfer kinetics. The Nyquist plots were fit with a modified Randles circuit comprising the solution resistance (*R*_s_), a charge-transfer resistance (*R*_ct_), and a constant-phase element (CPE) to account for nonideal double-layer behavior (Figure 5b). At the chosen DC bias, HES-CuTi exhibits the smallest *R*_ct_, consistent with its higher electronic conductivity arising from Cu/Ti incorporation, while HES-Co0.4 shows an intermediate *R*_ct_, plausibly aided by its relatively high Co content (both Co- and Cu-containing disulfides are known to be good electronic conductors). As expected, increasing the fraction of more conductive constituents shrinks the semicircle diameter, indicating a lower *R*_ct_ and faster interfacial charge transfer.

However, conductivity alone does not govern OER/ORR performance. Despite its comparatively larger *R*_ct_ under EIS conditions, HES-TM delivers the best catalytic activity (lowest η_10_, early onset, and steep polarization), showing that the density and quality of surface active sites and adsorbate binding energetics are decisive. In alkaline OER, strong metal–sulfur coordination can act as catalytic motifs: they promote OH^–^ adsorption, stabilize O*/*OOH intermediates, and facilitate O–O bond formation. The performance of the high-entropy sulfide catalysts can be explained by the electronic structure modification which can be referred from the XPS data shown in Figure 4. Most of the transition metals have both 2+ and 3+ oxidation states. Specifically, Co 2p spectra (Figure 4g–i) are dominated by the Co^3+^ state across all samples. A higher oxidation state has a higher oxidative potential to boost the OER activity, and Co^3+^ is widely recognized as a key active species for generating active oxyhydroxide intermediates.^7,39^ Since Co^3+^ is present in all samples, the variation in performance implies that the role of other cations and their coordination environment cannot be ignored.

We found that the addition of Cu and Ti in HES-CuTi resulted in a more reduced Fe state (higher Fe^2+^/Fe^3+^ ratio). It was found that the overpotential generally decreases with the maintenance of higher valence metal states, indicating that the high-valence cations effectively modulates the binding energy of oxygen intermediates. Furthermore, the effect of S_2_^2–^ and SO_*x*_ cannot be underestimated.^40,41^ As shown in Figure 4j–l, the oxidation of the sulfide surface induces the formation of SO_*x*_ in the samples. HES-TM, which exhibits the best bifunctional performance, shows a higher concentration of metal-coordinated disulfide and a specific modest SO_*x*_ layer compared to HES-CuTi. Although the interplay among metal centers results in new valence states, the role of sulfur species is critical as the anion environment tunes the electronic density of the metals. Here we have found that the S_2_^2–^/SO_*x*_ content correlates to the catalytic activity in a way that HES-TM, with its optimized sulfur coordination, outperforms HES-CuTi despite the latter’s lower charge transfer resistance. In other words, the electronic structural modification that reaches the aforementioned equilibrium active sites involves both the high-valence cations and the surface anions. The importance of this anion–cation synergy is seen where HES-CuTi shows worse performance due to its lower S_2_^2–^ fraction and reduced metal states, proving that conductivity alone is insufficient without the advantageous active-site chemistry. These defects and ligand-field environments likely tune *OH/*OOH binding toward optimal values, explaining why HES-TM outperforms more conductive compositions yet offers more catalytically competent sites.

The oxygen reduction reaction (ORR) activity of the HESs are shown in Figure 5c. Rotating-disk LSVs were recorded, and at a benchmark cathodic current density of *j* = −1 mA cm^–2^, the potentials (vs RHE) for HES-TM, HES-CuTi, and HES-Co0.4 are 0.69, 0.48, and 0.59 V, respectively. Because a more positive potential at a fixed cathodic current indicates better ORR activity, HES-TM outperforms the other sulfide electrocatalysts. Electron transfer numbers (*n*) and kinetic current densities (*J*_k_) were extracted using the Koutecký-Levich (K–L) analysis. To construct the K–L plots, LSV curves were collected between 1.20 and 0.14 V (vs RHE) at rotation rates of 400, 800, 1200, 1600, and 2000 rpm with a 5 mV s^–1^ scan rate using RDE setup (Figure S5). As the rotation rate increases, the diffusion layer thins and the mass-transport-limited current increases, consistent with Levich behavior. The resulting K–L plots indicate mixed pathways for all samples, yielding n-values between 2 and 4 (≈3 on average). The derived *J*_k_ values are also similar across compositions, suggesting comparable intrinsic ORR kinetics and consistent activity trends among the HESs.

Bifunctional performance was assessed using the bifunctional index (BI), defined here as the potential gap between the OER potential at 10 mA cm^–2^ and the ORR potential at −1 mA cm^–2^ (both vs RHE). The BI values for HES-TM, HES-CuTi, and HES-Co0.4 are 0.94, 1.19, and 1.16 V, respectively, with lower values indicating better bifunctionality. Importantly, BI is a useful predictor for rechargeable zinc-air battery performance: a lower BI typically correlates with a smaller charge–discharge voltage gap (Δ*E*) in full cells, reflecting reduced overpotential penalties at both oxygen electrodes and improved round-trip efficiency. Accordingly, the low BI of HES-TM suggests reduced polarization and superior zinc-air performance compared with HES-CuTi and HES-Co0.4.

### 3.4 Rechargeable Zinc-Air Battery Performance

Rechargeable Zn-air cells were assembled using a Zn-plate anode, a high-entropy sulfide (HES) air cathode, and 6 M KOH containing a 0.2 m Zn­(OAc)_2_ additive. The open-circuit voltage (OCV) of the HES-CuTi cell is slightly higher than the others, plausibly reflecting a more positive cathode mixed potential under air and/or lower internal polarization owing to the higher electronic conductivity contributed by Cu and Ti. Figure 6a shows the charge–discharge polarization curves. The cell with the HES-TM cathode exhibits a lower charging voltage and a higher discharge voltage than the HES-CuTi and HES-Co0.4 cells, indicating a smaller charge–discharge voltage gap (reduced polarization) and thus better rechargeability. Consistent with this, the HES-TM cathode delivers a peak power density of 88 mW cm^–2^ at 175 mA cm^–2^, surpassing HES-CuTi (73 mW cm^–2^). These results align with the lower bifunctional index (BI) of HES-TM, which is a useful predictor of zinc-air battery performance: a lower BI generally correlates with a smaller charge–discharge voltage gap in full cells.

**Figure 6:**
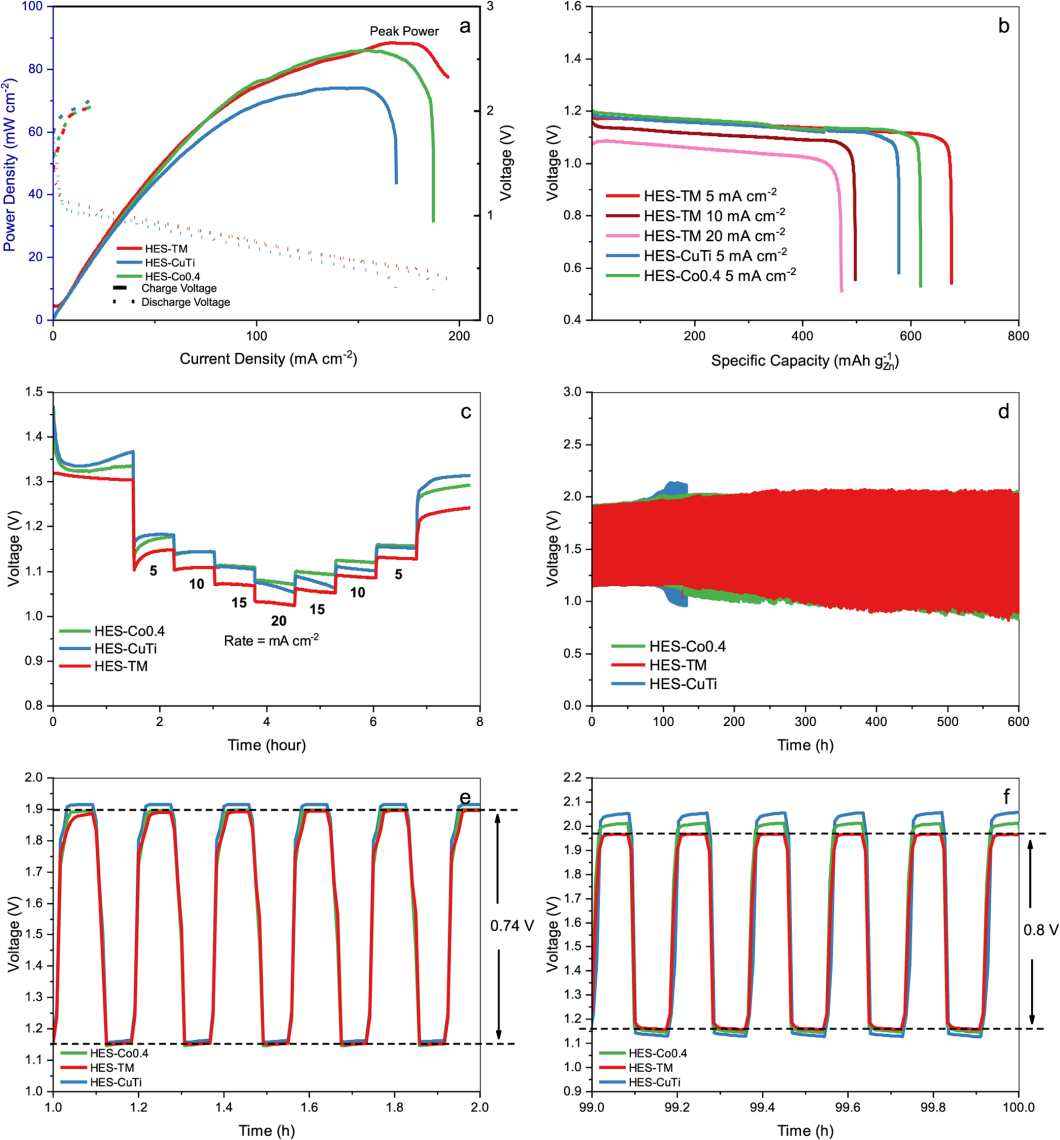
Zn-air batteries with HES-TM, HES-CuTi, and HES-Co0.4 electrocatalysts as air cathode (a) Charge and discharge polarization curves, and corresponding peak power density plots. (b) Specific capacities at 5, 10, and 20 mA cm^–2^ current densities, (c) rate capability study between 0 to 20 mA cm^–2^, (d) Cyclic charge–discharge curves at 5 mA cm^–2^, (e) durability performance in the first hour and (f) at 100 h of the cyclic charge–discharge.

Figure 6b summarizes the discharge capacities at current densities from 5 to 20 mA cm^–2^ for HES-TM, together with the capacities of HES-CuTi and HES-Co0.4 at 5 mA cm^–2^. The HES-TM-based Zn-air battery delivers a maximum discharge capacity of 675.9 mAh and a specific energy of 757 Wh kg^–1^. Notably, it exhibits excellent discharge capacity of 497 mAh and 472 mAh at 10 and 20 mA cm^–2^, respectively.

Figure 6c compares the rate capability and cycling stability of the HES-based Zn-air batteries. For the rate test, the discharge current density was stepped from 0 to 20 mA cm^–2^ and then back to 0 mA cm^–2^ while monitoring the cell voltage. As shown in Figure 6c, all HES-based cells exhibit robust rate performance and stable output during the step sequence. For example, the HES-TM cell maintains an output voltage of 1.242 V after 400 min, corresponding to 95.2% of its initial value (1.305 V), a decay of only 63 mV, indicating excellent stability under dynamic load conditions.

To assess electrochemical durability, galvanostatic charge–discharge cycling was performed at 5 mA cm^–2^. In the initial cycles, the Zn-air cell with the HES-TM cathode exhibits a charge voltage of 1.89 V and a discharge voltage of 1.15 V, giving a voltage gap Δ*E* = 0.74 V (Figure 6e). This gap is lower than that of many oxide-based bifunctional electrocatalysts and remains near this value for the first ∼100 h (Figure 6f). Thereafter, Δ*E* increases gradually, reaching ∼1.0 V after ∼25 days of continuous operation, which is still close to the bifunctional index (BI ≈ 0.94 V) inferred from half-cell metrics. By contrast, the HES-CuTi cell loses stability after ∼100 h, and the HES-Co0.4 cell shows a sharp increase in ΔE beyond the first 100 h. These results indicate that HES-TM affords superior long-term rechargeability in full Zn-air cells. To contextualize the practical performance of HES-TM, Table S2 compares its bifunctional metrics and battery performance against recently reported state-of-the-art non-noble metal electrocatalysts, including other high-entropy oxides, sulfides, and alloys. HES-TM exhibits a highly competitive OER potential of 1.540 V (at 10 mA cm^–2^), outperforming several comparable sulfide-based systems. While the peak power density (88 mW cm^–2^) is consistent with typical sulfide-based air cathodes, the most significant advantage of the entropy-engineered HES-TM is its exceptional durability. The battery achieves a cycle life of 600 h, which is 2- to 6-fold longer than the majority of the catalysts. This superior longevity confirms that the high-entropy lattice significantly enhances structural robustness against corrosion during long-term charge–discharge cycling.

The sustained performance of HES-TM is consistent with its lower BI and superior half-cell OER/ORR activity. As discussed above, HES-TM’s surface chemistry, stronger metal–sulfur coordination and modest SO*_x_* (oxy-sulfide) component, promote favorable S–OH/O*/*OOH interactions, while the mixed-valent transition-metal states (predominantly Co^3+^, mixed Ni^2+^/Ni^3+^, Fe^2+^/Fe^3+^) support redox flexibility. Together, these features lower overpotential penalties at both oxygen electrodes, yielding a smaller charge–discharge gap and enhanced cycling stability.

## 4 Conclusions

This work establishes high-entropy sulfides as an effective class of bifunctional oxygen electrocatalysts for zinc-air batteries. Structurally, all three HES compositions form single-phase pyrite with uniform multication distributions, providing a robust platform for tunable surface chemistry. Electrochemically, HES-TM outperforms HES-CuTi and HES-Co0.4 in both half-cell and full-cell tests: it combines low OER overpotential (310 mV at 10 mA cm^–2^) and higher ORR potential (0.69 V at −1 mA cm^–2^) with a favorable bifunctional index (0.94 V), translating to reduced charge–discharge polarization, 88 mW cm^–2^ peak power density, and high specific capacity/energy. Mechanistically, XPS indicates predominantly Co^3+^ with mixed Ni and Fe valence, and for HES-TM a larger S_2_^2–^ fraction plus a thin SO*_x_* layer; together these features imply stronger metal–sulfur coordination, and optimized adsorption energetics for *OH/*OOH, which better explain activity trends than electronic conductivity alone. Durability testing shows HES-TM maintains a low voltage gap (∼0.74 V initially; ∼1.0 V after ∼25 days), whereas HES-CuTi and HES-Co0.4 degrade more rapidly, underscoring the importance of surface defect chemistry and oxy-sulfide conditioning in sustaining bifunctional activity.

Beyond reporting strong ZAB metrics, this study provides design rules for HES catalysts: maximize S_2_^2–^-dominated surfaces with controlled SO*_x_* for coupled electron/ion transfer; exploit multication synergy to tune local ligand fields and defect formation; and recognize that high conductivity is beneficial but not predictive without proper active-site chemistry. Finally, by repurposing materials initially developed for thermal batteries, we offer a general framework for translating high-temperature, multicomponent chemistries into room-temperature, aqueous ZABs, pointing to scalable, cost-effective cathodes for next-generation energy storage.

## Supplementary Material



## Notes

Rietveld-refined XRD patterns of the synthesized high-entropy spinels; SEM images together with EDS elemental distribution maps of HES-CuTi and HES-Co0.4, while SEM images and W elemental distribution for HES-TM, HES-CuTi, and HES-Co0.4; XPS core-level spectra of Cr 2p, Mn 2p, Cu 2p, and Ti 2p for all HES samples; Linear sweep voltammetry curves recorded at different rotation speeds (400–2000 rpm); The ICP-OES results along with the corresponding weight and mole percentages of the elements; A comparison of the bifunctional oxygen electrocatalytic activity and rechargeable zinc–air battery performance of the HESs (PDF)

## References

[1] Nazir G., Rehman A., Lee J. H., Kim C. H., Gautam J., Heo K., Hussain S., Ikram M., AlObaid A. A., Lee S. Y., Park S. J. (2024). A Review of Rechargeable Zinc–Air Batteries:
Recent Progress and Future Perspectives. Nano-Micro
Lett..

[2] Lee S., Choi J., Kim M., Park J., Park M., Cho J. (2022). Material Design and
Surface Chemistry for Advanced Rechargeable Zinc-Air
Batteries. Chem. Sci..

[3] Lv X. W., Wang Z., Lai Z., Liu Y., Ma T., Geng J., Yuan Z. Y. (2024). Rechargeable Zinc–Air
Batteries:
Advances, Challenges, and Prospects. Small.

[4] Zhuang B., Xu N., Xu X., Dai L., Wang Y., Wang M., Wu K., Qiao J. (2025). Hierarchically Mesoporous Fe-N-C Single-Atom Catalysts
for Efficient Oxygen Electrocatalysis in Rechargeable Zinc-Air Batteries. Mater. Rep.: Energy.

[5] Zhang Y., Xu N., Gong B., Ye X., Yang Y., Wang Z., Zhuang B., Wang M., Yang W., Liu G., Lee J. K., Qiao J. (2025). A Visible-Light-Driven
CoS2/CuS@CNT-C3N4
Photocatalyst for High-Performance Rechargeable Zinc-Air Batteries
beyond 500 MW Cm^–2^. Chin.
J. Catal..

[6] Huang Y., Wang Y., Tang C., Wang J., Zhang Q., Wang Y., Zhang J. (2019). Atomic Modulation and Structure Design
of Carbons for Bifunctional Electrocatalysis in Metal–Air Batteries. Adv. Mater..

[7] Nguyen T. X., Su Y., Lin C., Ting J. (2021). Self-Reconstruction of Sulfate-Containing
High Entropy Sulfide for Exceptionally High-Performance Oxygen Evolution
Reaction Electrocatalyst. Adv. Funct Mater..

[8] Sun Q., Du H.-H., Sun T.-J., Li D.-T., Cheng M., Liang J., Li H.-X., Tao Z.-L. (2024). Sorbitol-Electrolyte-Additive
Based Reversible Zinc Electrochemistry. J. Electrochem..

[9] Cheng X., Dong J., Yang H., Li X., Zhao X., Chen B., Zhang Y., Liu M., Wang J., Lin H. (2025). Tailoring Solvation Sheath for Rechargeable
Zinc-Ion Batteries: Progress
and Prospect. Mater. Rep.: Energy.

[10] Wang X. X., Yang X., Liu H., Han T., Hu J., Li H., Wu G. (2022). Air Electrodes for
Flexible and Rechargeable Zn–Air
Batteries. Small Struct..

[11] Wang S., Lu A., Zhong C. J. (2021). Hydrogen Production from Water Electrolysis: Role of
Catalysts. Nano Convergence.

[12] McCrory C. C. L., Jung S., Peters J. C., Jaramillo T. F. (2013). Benchmarking
Heterogeneous Electrocatalysts for the Oxygen Evolution Reaction. J. Am. Chem. Soc..

[13] Wei C., Rao R. R., Peng J., Huang B., Stephens I. E. L., Risch M., Xu Z. J., Shao-Horn Y. (2019). Recommended
Practices and Benchmark Activity for Hydrogen and Oxygen Electrocatalysis
in Water Splitting and Fuel Cells. Adv. Mater..

[14] Erdil T., Ozgur C., Geyikci U., Lokcu E., Toparli C. (2024). Earth-Abundant
Divalent Cation High-Entropy Spinel Ferrites as Bifunctional Electrocatalysts
for Oxygen Evolution and Reduction Reactions. ACS Appl. Energy Mater..

[15] Bayraktar D. O., Lökçü E., Ozgur C., Erdil T., Toparli C. (2022). Effect of Synthesis
Environment on the Electrochemical
Properties of (FeMnCrCoZn)­3O4 High-Entropy Oxides for Li-Ion Batteries. Int. J. Energy Res..

[16] Ozgur C., Erdil T., Geyikci U., Okuyucu C., Lokcu E., Kalay Y. E., Toparli C. (2024). Engineering
Oxygen Vacancies in (FeCrCoMnZn)­3O4-δ
High Entropy Spinel Oxides Through Altering Fabrication Atmosphere
for High-Performance Rechargeable Zinc-Air Batteries. Global Challenges.

[17] Fracchia M., Coduri M., Ghigna P., Anselmi-Tamburini U. (2024). Phase Stability
of High Entropy Oxides: A Critical Review. J.
Eur. Ceram Soc..

[18] Rost C. M., Sachet E., Borman T., Moballegh A., Dickey E. C., Hou D., Jones J. L., Curtarolo S., Maria J. P. (2015). Entropy-Stabilized Oxides. Nat.
Commun..

[19] Geyikci U., Erdil T., Ozgur C., Toparli C. (2025). Optimizing Cation Synergy
in High Entropy Oxides for Superior Bifunctional Oxygen Electrocatalysis. Electrochim. Acta.

[20] Coskuner A. B., Erdil T., Ozgur C., Geyikci U., Toparli C. (2025). Tuning the
Lattice Strain through Manipulating Crystal Structure of High Entropy
Oxides Enhances Electrocatalytic Performance. Mater. Res. Bull..

[21] Erdil T., Toparli C. (2023). B-Site Effect on High-Entropy
Perovskite Oxide as a
Bifunctional Electrocatalyst for Rechargeable Zinc-Air Batteries. ACS Appl. Energy Mater..

[22] Ozgur C., Erdil T., Geyikci U., Yildiz I., Lokcu E., Toparli C. (2024). B-Site Doping Boosts
the OER and ORR Performance of
Double Perovskite Oxide as Air Cathode for Zinc-Air Batteries. ChemPhysChem.

[23] Erdil T., Lokcu E., Yildiz I., Okuyucu C., Kalay Y. E., Toparli C. (2022). Facile Synthesis and Origin of Enhanced Electrochemical
Oxygen Evolution Reaction Performance of 2H-Hexagonal Ba2CoMnO6-Δas
a New Member in Double Perovskite Oxides. ACS
Omega.

[24] Guo S., Wu J., Chen H., Huan D., Wang H., Wang D., Hou D., Li X. (2025). ZIF-67-Derived Cation
Regulation of Metal Sulfides
for Boosting Oxygen Evolution Activity. ACS
Appl. Energy Mater..

[25] Lee C. W., Sathiyanarayanan K., Eom S. W., Yun M. S. (2006). Novel Alloys to
Improve the Electrochemical Behavior of Zinc Anodes for Zinc/Air Battery. J. Power Sources.

[26] Cui Y., Zhang W., Li Y., Guo Y., Hanzawa N., Yamauchi Y., Sugahara Y. (2025). Hollow-Structured High-Entropy
Metal
Sulfide (Co,Ni,Cu,Zn,Mn) 3 S 4 As An Efficient Supercapacitor Electrode. ACS Appl. Energy Mater..

[27] Wang Y., Liu H., Chen J., Han K., Xia T., Yang H., Yuan X., Zhao Y. (2025). Facile Synthesis of High-Entropy
Sulfide Catalyst for Oxygen Evolution Reaction by a Two-step Route. J. Power Sources.

[28] Moradi M., Hasanvandian F., Bahadoran A., Shokri A., Zerangnasrabad S., Kakavandi B. (2022). New High-Entropy Transition-Metal Sulfide Nanoparticles
for Electrochemical Oxygen Evolution Reaction. Electrochim. Acta.

[29] Li H., Ling L., Li S., Gao F., Lu Q. (2023). High Entropy
Materials—Emerging Nanomaterials for Electrocatalysis. Energy Adv..

[30] Chen J., Wang K., Liu Z., Sun X., Zhang X., Lei F., Wan X., Xie J., Tang B. (2024). Sulfurization-Induced
Lattice Disordering in High-Entropy Catalyst for Promoted Bifunctional
Electro-Oxidation Behavior. Chem. Eng. J..

[31] Huo W., Wang S., Dominguez-Gutierrez F.
J., Ren K., Kurpaska Ł., Fang F., Papanikolaou S., Kim H. S., Jiang J. (2023). High-Entropy Materials for Electrocatalytic
Applications: A Review of First Principles Modeling and Simulations. Mater. Res. Lett..

[32] Zhao Y., You J., Wang Z., Liu G., Huang X., Duan M., Zhang H. (2024). High-Entropy FeCoNiCuAlV
Sulfide as an Efficient and Reliable Electrocatalyst
for Oxygen Evolution Reaction. Int. J. Hydrogen
Energy.

[33] Zhu R., Han M., Dong L., Tian X., Sun F., Liu R., Zang J., Wang Y. (2025). MoS2-Wrapped NiFeCoCrSn High-Entropy-Sulfide
Catalyst for Efficient and Stable Overall Water Splitting. J. Alloys Compd..

[34] Xiao W., Li Y., Elgendy A., Duran E. C., Buckingham M. A., Spencer B. F., Han B., Alam F., Zhong X., Cartmell S. H., Cernik R. J., Eggeman A. S., Dryfe R. A. W., Lewis D. J. (2023). Synthesis of High Entropy and Entropy-Stabilized
Metal
Sulfides and Their Evaluation as Hydrogen Evolution Electrocatalysts. Chem. Mater..

[35] Biesinger M. C., Payne B. P., Hart B. R., Grosvenor A. P., McIntryre N. S., Lau L. W., Smart R. S. (2008). Quantitative
Chemical
State XPS Analysis of First Row Transition Metals, Oxides and Hydroxides. J. Phys. Conf. Ser..

[36] Lin L., Wang K., Sarkar A., Njel C., Karkera G., Wang Q., Azmi R., Fichtner M., Hahn H., Schweidler S., Breitung B. (2022). High-Entropy Sulfides as Electrode
Materials for Li-Ion Batteries. Adv. Energy
Mater..

[37] Jiang J., Lu S., Gao H., Zhang X., Yu H.-Q. (2016). Ternary FeNiS2 Ultrathin
Nanosheets as an Electrocatalyst for Both Oxygen Evolution and Reduction
Reactions. Nano Energy.

[38] Ai G., Hu Q., Zhang L., Dai K., Wang J., Xu Z., Huang Y., Zhang B., Li D., Zhang T., Liu G., Mao W. (2019). Investigation of the
Nanocrystal CoS 2 Embedded in
3D Honeycomb-like Graphitic Carbon with a Synergistic Effect for High-Performance
Lithium Sulfur Batteries. ACS Appl. Mater. Interfaces.

[39] Bergmann A., Martinez-Moreno E., Teschner D., Chernev P., Gliech M., de Araújo J. F., Reier T., Dau H., Strasser P. (2015). Reversible
Amorphization and the Catalytically Active State of Crystalline Co3O4
during Oxygen Evolution. Nat. Commun..

[40] Wang T., Nam G., Jin Y., Wang X., Ren P., Kim M. G., Liang J., Wen X., Jang H., Han J., Huang Y., Li Q., Cho J. (2018). NiFe (Oxy) Hydroxides
Derived from NiFe Disulfides as an Efficient Oxygen Evolution Catalyst
for Rechargeable Zn–Air Batteries: The Effect of Surface S
Residues. Adv. Mater..

[41] Gao M., He L., Guo Z.-Y., Yuan Y.-R., Li W.-W. (2020). Sulfate-Functionalized
Nickel Hydroxide Nanobelts for Sustained Oxygen Evolution. ACS Appl. Mater. Interfaces.

