# Correction to “Pyrite High-Entropy Sulfides for Bifunctional Oxygen Electrocatalysis and High-Performance Zinc-Air Batteries”

**DOI:** 10.1021/acsomega.6c03769

**Published:** 2026-05-25

**Authors:** Tuncay Erdil, Nazlican Uysal, Zeynep Ilgın Yüceer, Cem Asci, Cagla Ozgur, Uygar Geyikci, Cigdem Toparli

**Affiliations:** † Department of Metallurgical and Materials Engineering, 52984 Middle East Technical University, 06800, Ankara, Türkiye; ‡ Battery Technologies Division, TUBITAK SAGE, 06800, Ankara, Türkiye; § Energy Storage Materials and Devices Research Center (ENDAM), Middle East Technical University, Ankara 06800, Türkiye

We are issuing a correction to add an author and their affiliation to the original manuscript. The author Cem Asci has been added to the author list, and his affiliation has been included. Changes are reflected in the Author Information with this Correction. We apologize for any inconvenience it may have caused.

