# Supplementary material for: Pyrite High-Entropy Sulfides for Bifunctional Oxygen Electrocatalysis and High-Performance Zinc-Air Batteries
Source: ACS Omega. 2026 Feb 19;11(12):19345–55. doi: 10.1021/acsomega.5c12740 (PMC13044606; doi:10.1021/acsomega.5c12740)
Supplement: Supplementary file 1 [file ao5c12740_si_001.pdf]

Pyrite High-Entropy Sulfides for Bifunctional Oxygen Electrocatalysis and High-Performance Zinc-Air Batteries

Tuncay Erdil[1], Nazlican Uysal[1], Zeynep Ilgın Yüceer[1], Cagla Ozgur[1], Uygar Geyikci[1] and Cigdem Toparli[1,2*]

[1]Department of Metallurgical and Materials Engineering, Middle East Technical University, 06800, Ankara, Türkiye

[2]Energy Storage Materials and Devices Research Center (ENDAM), Middle East Technical University, Ankara 06800, Türkiye

List of Figures

Figure S1. Rietveld refined XRD patterns of HESs (a) HES-TM, (b) HES-CuTi, (c) HES-Co0.4.

Figure S2. SEM images and EDS distribution of HESs (a) HES-CuTi, (b) HES-Co0.4.

Figure S3. SEM images and EDS W distribution of HESs (a) HES-TM, (b) HES-CuTi, (c) HES-Co0.4.

Figure S4. XPS core level spectra of (a) Cr 2p, (b) Mn 2p, (c) Cu 2p and (d) Ti 2p for HESs.

Figure S5. Linear Sweep Voltammetry of HESs at different rotation speeds (400, 800, 1200, 1600, 2000 rpm)

Table S1. ICP-OES results and weight/mole percentage of elements in HESs.

Table S2. Comparison of the bifunctional oxygen electrocatalytic activity and rechargeable Zn-air battery performance.

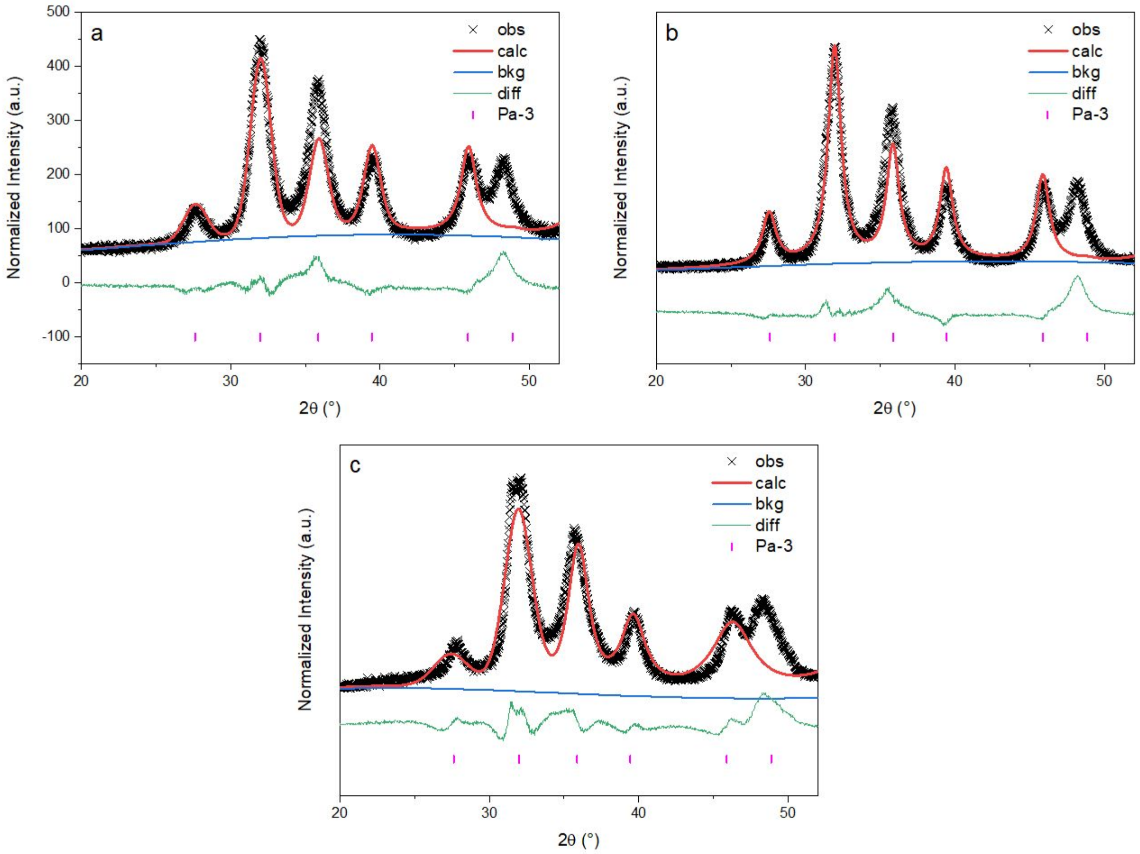

Figure S1. Rietveld refined XRD patterns of HESs (a) HES-TM, (b) HES-CuTi, (c) HES-Co0.4.

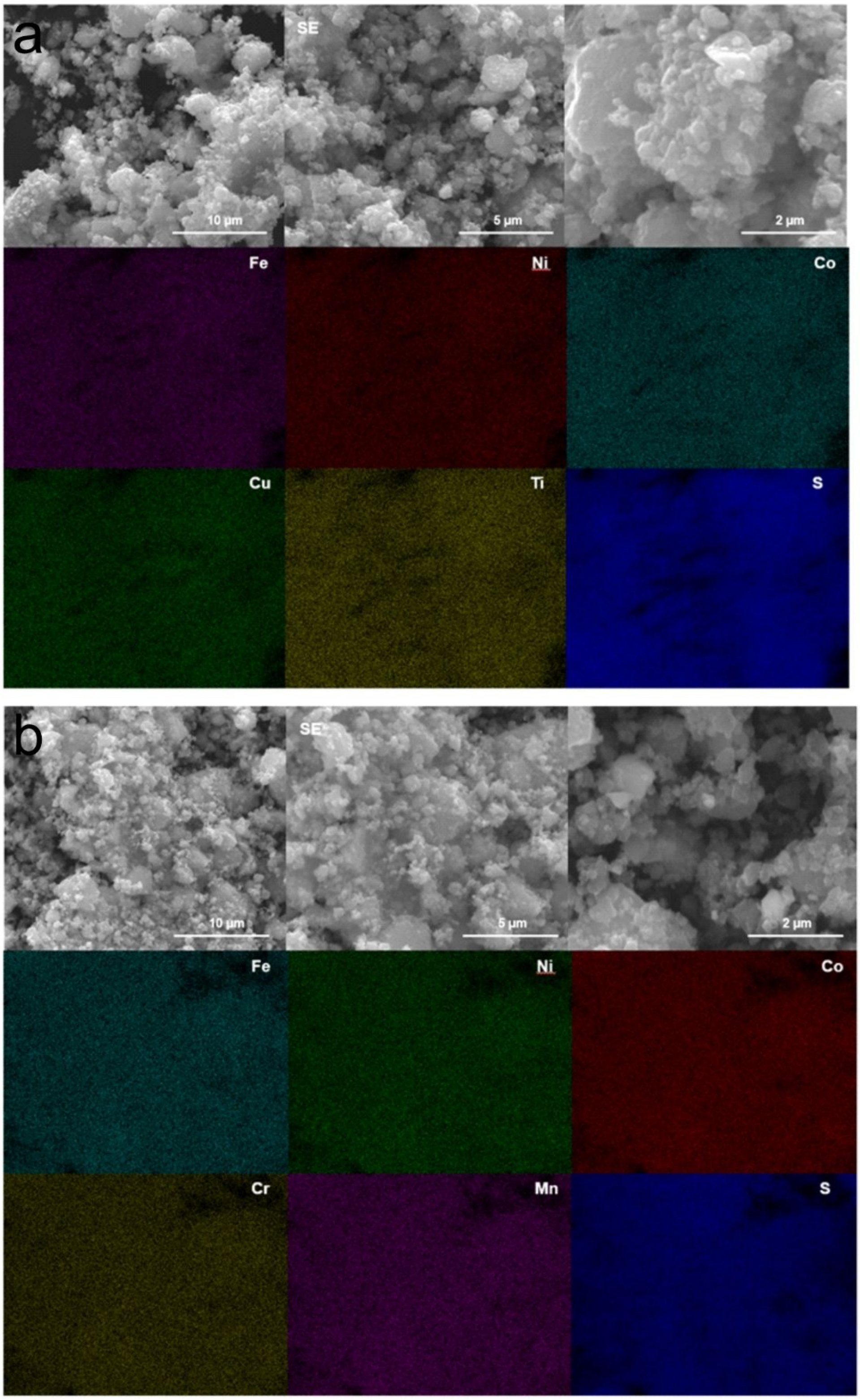

Figure S2. SEM images and EDS distribution of HESs (a) HES-CuTi, (b) HES-Co0.4.↔

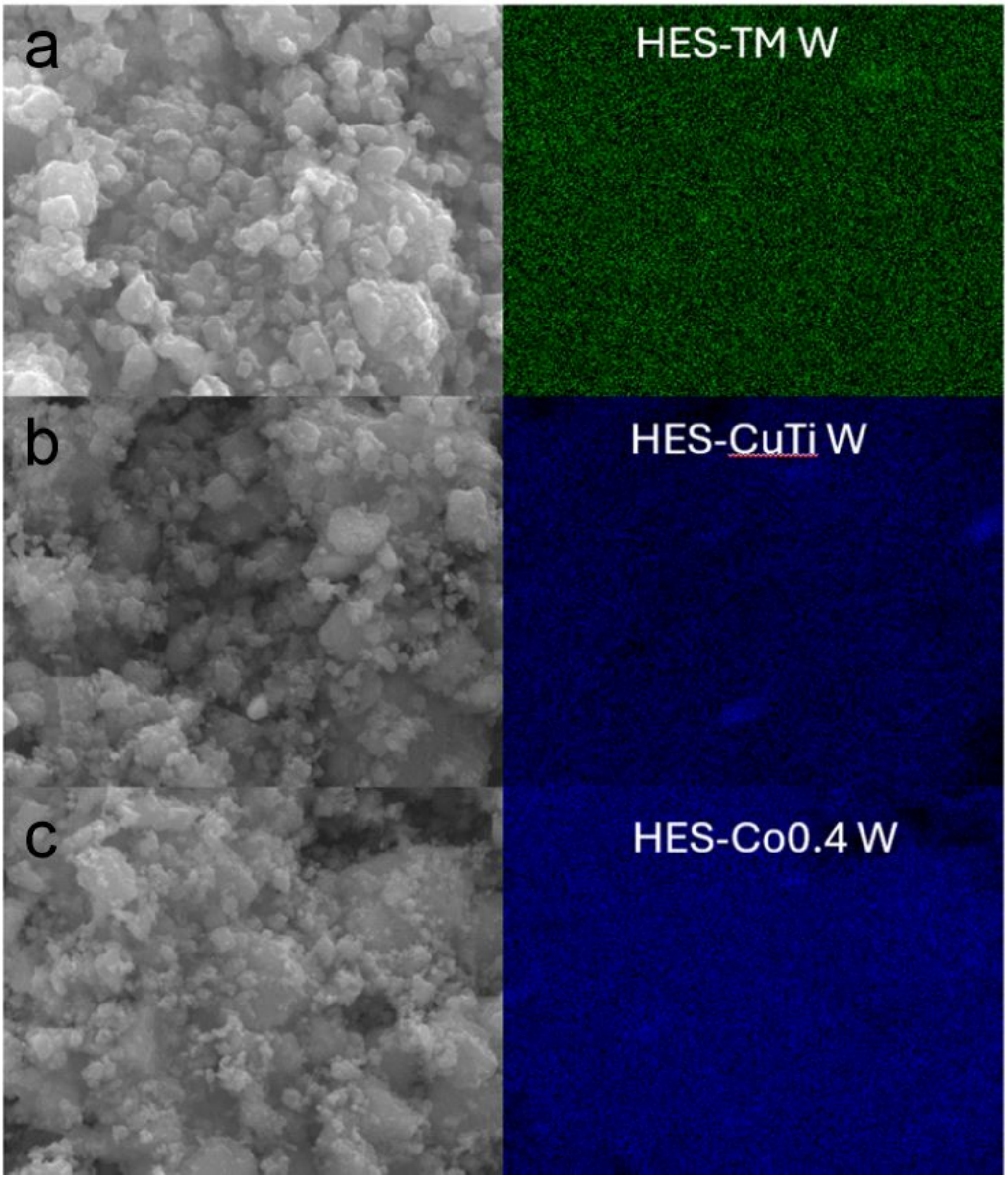

Figure S3. SEM images and EDS distribution of HESs (a) HES-TM, (b) HES-CuTi, (c) HES-Co0.4.

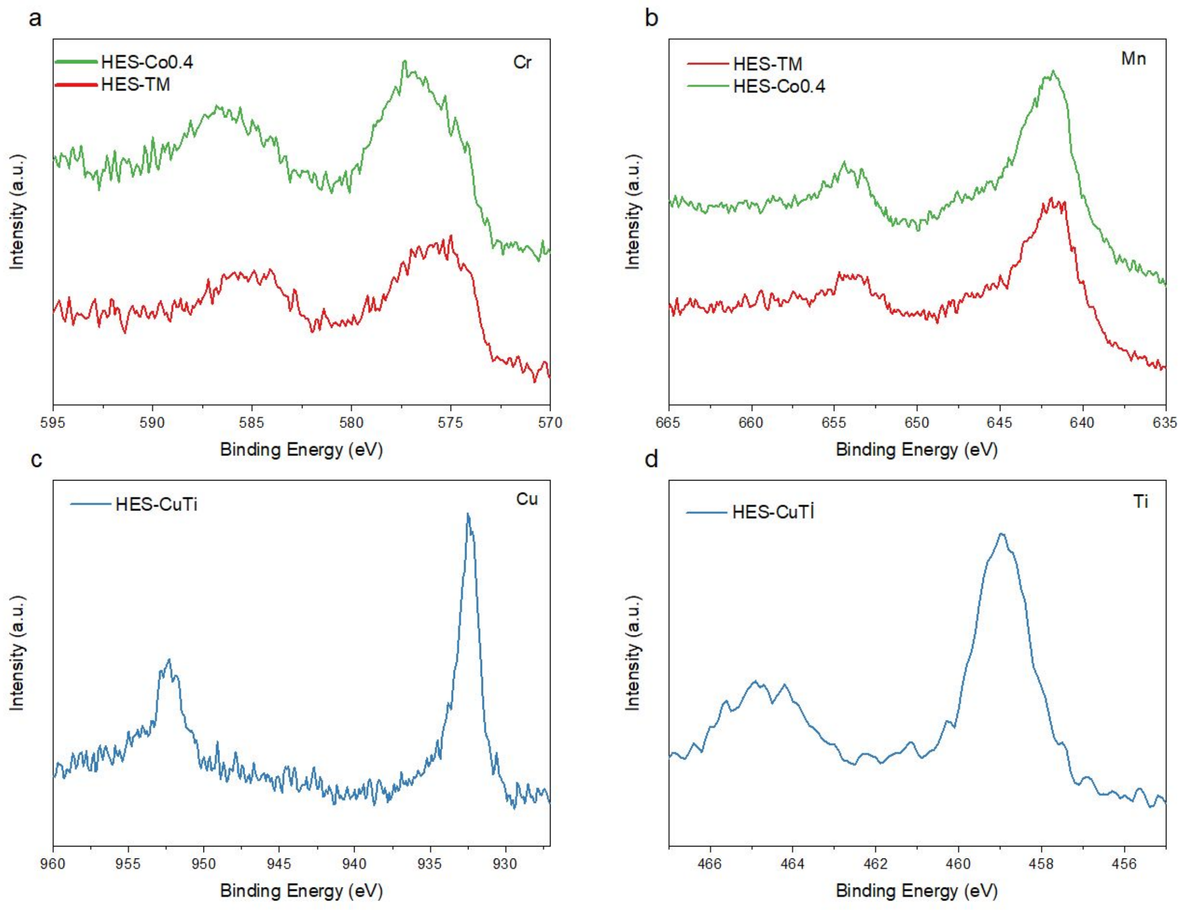

Figure S4. XPS core level spectra of (a) Cr 2p, (b) Mn 2p, (c) Cu 2p and (d) Ti 2p for HESs.

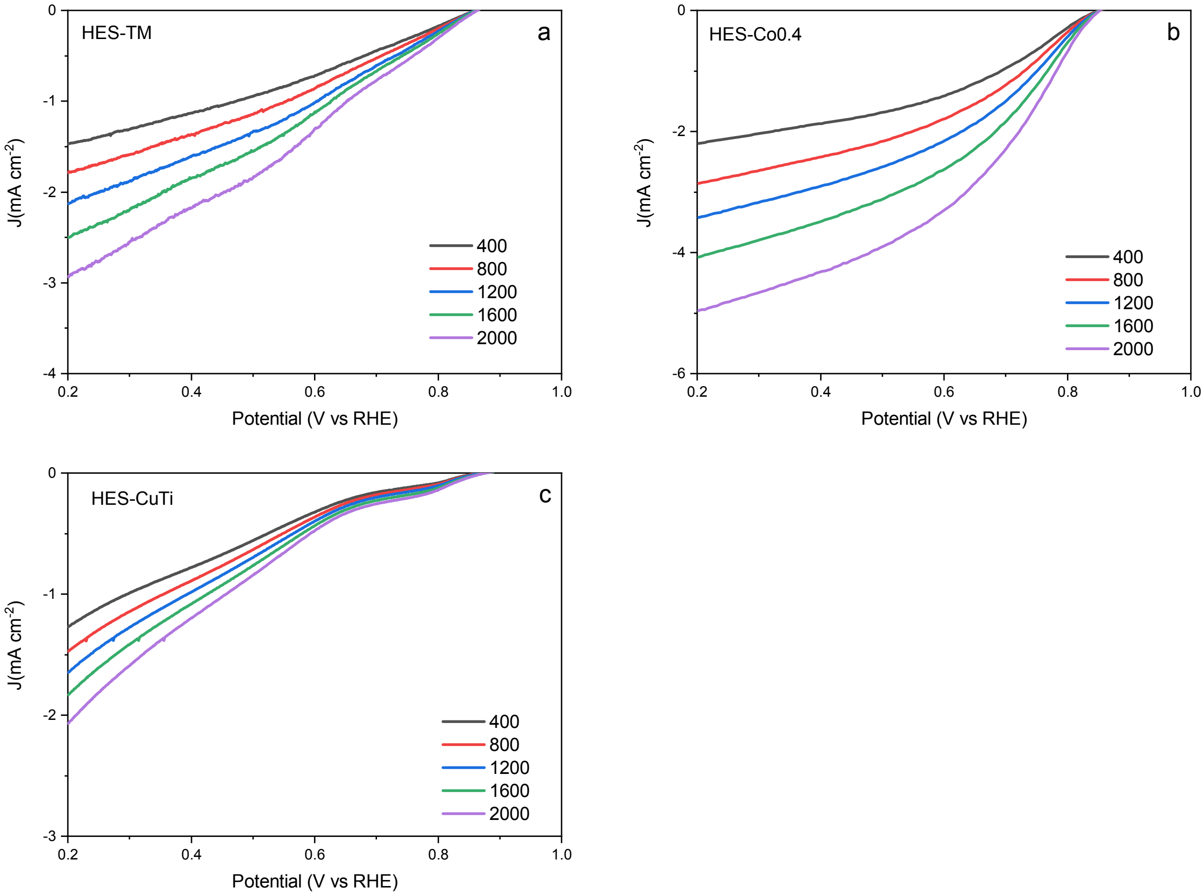

Figure S5. Linear Sweep Voltammetry at different rotation speeds (400, 800, 1200, 1600, 2000 rpm) of (a) HES-TM (b) HES-Co0.4 (c) HES-CuTi,

Table S1. ICP-OES results and weight/mole percentage of elements in HESs.

	**HES-TM**		**HES-CuTi**		**HES-Co0.4**	
	**mol%**	**wt.%**	**mol%**	**wt.%**	**mol%**	**wt.%**
**Fe (wt%)**	0.19 ±0.02	9.00 ±0.1	0.20 ±0.02	9.30 ±0.1	0.15 ±0.02	6.91 ±0.1
**Co (wt%)**	0.23 ±0.02	11.28 ±0.1	0.24 ±0.02	11.66 ±0.1	0.40 ±0.02	19.29 ±0.1
**Mn (wt%)**	0.19 ±0.02	8.52 ±0.1	-		0.15 ±0.02	6.91 ±0.1
**Cr (wt%)**	0.20 ±0.02	8.52 ±0.1	-		0.15 ±0.02	6.62 ±0.1
**Ni (wt%)**	0.19 ±0.02	9.24 ±0.1	0.20 ±0.02	9.55 ±0.1	0.17 ±0.02	8.35 ±0.1
**Cu (wt%)**	-		0.21 ±0.02	11.04 ±0.1	-	
**Ti (wt%)**	-		0.19 ±0.02	7.44 ±0.1	-	
**S (wt%)**	1.98 ±0.07	52.8 ±1.0	1.95 ±0.07	52.08 ±1.0	1.94 ±0.07	51.84 ±1.0

Table S2. Comparison of the bifunctional oxygen electrocatalytic activity and rechargeable Zn-air battery performance.

Catalyst	OER[@10 mA cm^{-2}] (V)	ORR @ $E_{1/2}$ (V)	Power Density (mW cm^{-2})	Cycle Life (h)	Reference
**HES-TM**	1.540	0.584	88	600	This work
**HES-Co0.4**	1.670	0.518	85	600	This work
**HES-CuTi**	1.755	0.443	73	100	This work
**Sr(FeCoNiMo)1/4O3−δ**	1.531	0.640	96.6	150	1
**HEPBA/CNT-800**	1.560	0.770	71	40	2
**Fe0.03CuCo2S4**	1.560	0.800	82	75	3
**(FeCoNiCrCu/CNFs)**	1.470	0.780	87	75	4
**(FeCrCoMnZn)3O4-δ**	1.559	-	102	300	5
**FeP-HESOs/C**	1.516	0.916	179.3	180	6
**3DOM Co9S8/Ni3S4**	1.564	0.770	77.88	100	7
**N-CuS**	1.510	0.790	101	60	8
**Fe1.5NiS0.5-TLCs-800**	1.500	0.840	152.6	160	9
**(AlCoFeMoCr)3O4**	1.490	0.870	132	200	10
**Co9S8/Co1-xS/WS2**	1.580	0.740	52.2	100	11
**Fe-NiS2/CoS2**	1.495	0.883	80	200	12
**CrMnFeCoNi**	1.495	0.761	116.5	240	13
**HESOx/OLCAT**	1.530	0.800	152	316	14

References

(1) Li, K.; Gao, J.; Han, X.; Shao, Q.; Lü, Z. Novel Medium Entropy Perovskite Oxide Sr(FeCoNiMo)1/4O3− for Zinc-Air Battery Cathode. *Journal of Energy Chemistry* 2024, *96*, 669–678. https://doi.org/10.1016/j.jechem.2024.05.030.

(2) Tanmathusorachai, W.; Aulia, S.; Rinawati, M.; Chang, L.-Y.; Chang, C.-Y.; Huang, W.-H.; Lin, M.-H.; Su, W.-N.; Yuliarto, B.; Yeh, M.-H. High-Entropy Prussian Blue Analogue Derived Heterostructure Nanoparticles as Bifunctional Oxygen Conversion Electrocatalysts for the Rechargeable Zinc–Air Battery. *ACS Appl. Mater. Interfaces* 2024, *16* (45), 62022–62032. https://doi.org/10.1021/acsami.4c13387.

(3) Sharma, R.; Rawat, A.; Singh, A.; Merlin johny, J.; Gautam, A.; Parui, J.; Das, T.; Halder, A. Fe-Doped CuCo 2 S 4 Thiospinel as a High-Performance Oxygen Electrocatalyst for Rechargeable All-Solid-State Zinc–Air Batteries. *J. Mater. Chem. A Mater.* 2026, *14* (4), 2218–2234. https://doi.org/10.1039/D5TA07350B.

(4) Xia, L.; Dai, P.; Qu, C.; Yang, Z.; Zhen, H.; Wang, K.; Wu, M. High-Entropy Alloy Self-Supporting Bifunctional Electrocatalysts with Exceptional Performance for Flexible Zinc–Air Batteries. *J. Mater. Chem. A Mater.* 2025, *13* (45), 39382–39392. https://doi.org/10.1039/D5TA04663G.

(5) Ozgur, C.; Erdil, T.; Geyikci, U.; Okuyucu, C.; Lokcu, E.; Kalay, Y. E.; Toparli, C. Engineering Oxygen Vacancies in (FeCrCoMnZn) 3 O 4-δ High Entropy Spinel Oxides Through Altering Fabrication Atmosphere for High-Performance Rechargeable Zinc-Air Batteries. *Global Challenges* 2024, *8* (1). https://doi.org/10.1002/gch2.202300199.

(6) Yang, B.; Bao, C.; Zhai, L.; Xiang, Z. High-Entropy Spinel Oxides Coupling with Covalent Organic Polymers as Bifunctional Oxygen Electrocatalysts for Zinc-Air Batteries. *Chemical Engineering Journal* 2025, *515*, 163757. https://doi.org/10.1016/j.cej.2025.163757.

(7) Zhang, Y.; Li, J.; Wang, H.; Zhang, K.; Liu, S.; Liu, G. Optimizing Cobalt/Nickle Sulfide Heterojunction Interface with Ordered Porous Framework for Efficient Rechargeable Zinc-Air Batteries. *J. Alloys Compd.* 2025, *1010*, 177416. https://doi.org/10.1016/j.jallcom.2024.177416.

(8) Jung, D. H.; Park, Y. H.; Kim, D. W.; Choi, J. H.; Cho, S.; Kim, K.; Park, D. G.; Han, B.; Kang, J. K. Serrated Leaf-Like N-Doped Copper Sulfide Enabling Bifunctional Oxygen Reduction/Evolution via Dual-Mode Cathode Reactions for High Energy Density and Cycle Stability in Zinc–Air Batteries. *Advanced Science* 2025, *12* (20). https://doi.org/10.1002/advs.202413425.

(9) Zhang, Y.; Tian, Y.; Han, Y.; Wang, X.; Ma, Z. Fabrication of N, S Co-Doped Lignin-Based Hierarchical Porous Carbon Nanocages Loaded with Binary Metal Sulfides as High-

Performance ORR/OER Cathode Materials for Zn-Air Batteries. *J. Energy Storage* 2025, *114*, 115822. https://doi.org/10.1016/j.est.2025.115822.

(10) Jin, Z.; Lyu, J.; Zhao, Y.-L.; Li, H.; Chen, Z.; Lin, X.; Xie, G.; Liu, X.; Kai, J.-J.; Qiu, H.-J. Top–Down Synthesis of Noble Metal Particles on High-Entropy Oxide Supports for Electrocatalysis. *Chemistry of Materials* 2021, *33* (5), 1771–1780. https://doi.org/10.1021/acs.chemmater.0c04695.

(11) Fan, B.; Lei, A.; Zou, Y.; Liu, M.; Zhao, L.; Jin, B. Three-Dimensional Nanoflower-like Transition Metal Sulfide Heterostructures (Co9S8/Co1-XS /WS2) as Efficient Bifunctional Oxygen Electrocatalysts for Zn-Air Batteries. *Electrochim. Acta* 2025, *512*, 145467. https://doi.org/10.1016/j.electacta.2024.145467.

(12) Lu, Z.; Li, G.; Sheng, K.; Wu, T.; Luo, W.; Zhou, H.; Yi, Q. Oxygen Evolution and Oxygen Reduction Catalyzed by Fe-Co-Ni Ternary Metal Sulfides for Rechargeable Zinc-Air Battery. *J. Alloys Compd.* 2025, *1039*, 183119. https://doi.org/10.1016/j.jallcom.2025.183119.

(13) He, R.; Yang, L.; Zhang, Y.; Wang, X.; Lee, S.; Zhang, T.; Li, L.; Liang, Z.; Chen, J.; Li, J.; Ostovari Moghaddam, A.; Llorca, J.; Ibáñez, M.; Arbiol, J.; Xu, Y.; Cabot, A. A CrMnFeCoNi High Entropy Alloy Boosting Oxygen Evolution/Reduction Reactions and Zinc-Air Battery Performance. *Energy Storage Mater.* 2023, *58*, 287–298. https://doi.org/10.1016/j.ensm.2023.03.022.

(14) Mongwe, A.; Haruna, A. B.; Gaolatlhe, L.; Soto, J.; Shi, Z.; Mwonga, P. V.; Yang, X.-Y.; Muller, D. A.; Abruña, H. D.; Ozoemena, K. I. Defect-Engineered High-Entropy Spinel Oxide@Onion-Like Carbon Catalysts for High-Areal-Energy Rechargeable Zinc–Air Batteries. *Energy & Fuels* 2025, *39* (27), 13105–13119. https://doi.org/10.1021/acs.energyfuels.5c02012.
